# Ectopic Hidradenitis Suppurativa: Case Report and Review of Literature

**DOI:** 10.7759/cureus.12966

**Published:** 2021-01-28

**Authors:** Nikolas Gutierrez, Philip R Cohen

**Affiliations:** 1 General Practice, 1st Marine Division, 1st Combat Engineer Battalion, Camp Pendleton, USA; 2 Dermatology, San Diego Family Dermatology, National City, USA

**Keywords:** sinus, adalimumab, apocrine, tract, atypical, ectopic, ectopic hidradenitis suppurativa, hidradenitis, suppurative, sinus tracts

## Abstract

Hidradenitis suppurativa is a chronic, recurrent follicular-based inflammatory condition classically occurring in apocrine-rich areas; commonly affected areas include the anogenital, axillary, inframammary, and inguinal regions. Infrequently, hidradenitis suppurativa can occur in locations where apocrine glands are scant or absent; in this setting, it has been referred to as ectopic hidradenitis suppurativa. The case of a 59-year-old man with ectopic hidradenitis suppurativa on his right posterior thigh is described. The postulated pathogenesis, treatment modalities, and various reported locations of ectopic hidradenitis suppurativa are reviewed.

## Introduction

Hidradenitis suppurativa is a follicular-based inflammatory disease defined by its chronicity, recurrence, and severe pain. Lesions typically first appear after puberty and classically present as painful, deep-seated abscesses with sinus tracts in areas where apocrine glands are located. Most commonly, lesions occur in the anogenital, axillary, inframammary, and inguinal regions [1,2].

However, less frequently, lesions may also occur in areas where apocrine glands are scant or absent. Previously reported atypical areas include the abdomen [3], amputation stump [4,5], caesarian scar [6], chest [7], dorsal foot [8], eyelids [9,10], knees [11], retroauricular [12], and scalp [13]. When lesions appear in these less common areas, the condition has been referred to as either atypical or ectopic hidradenitis suppurativa.

The case of a 59-year-old Caucasian man who developed ectopic hidradenitis suppurativa on the right posterior thigh is described. In addition, the characteristics of previously reported individuals with ectopic hidradenitis suppurativa are reviewed. Also, the speculated pathogenesis and various treatment modalities for hidradenitis suppurativa are discussed.

## Case presentation

A 59-year-old Caucasian man with a past medical history of thyroid cancer that was treated with radioactive ablation subsequently maintained on levothyroxine and hypertension presented with a painful, enlarging lesion on his right posterior thigh. The lesion initially appeared as a papule that progressed to a pustule from which purulent fluid could be expressed. He also had a prior history of methicillin-susceptible *Staphylococcus aureus* (MSSA) infection that resolved after treatment with oral cephalexin.

**Figure 1:**
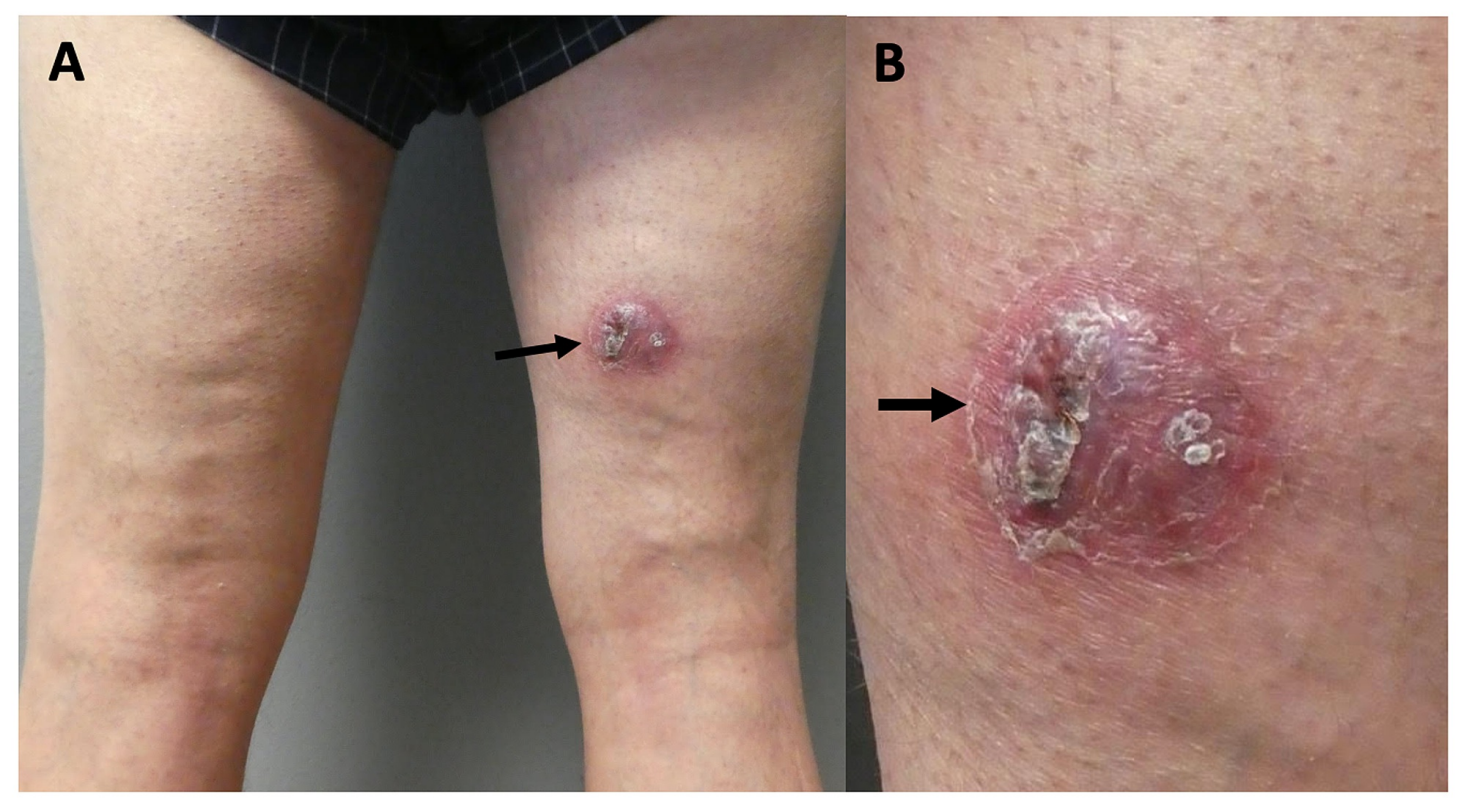
Initial clinical presentation of ectopic hidradenitis suppurativa. Distant (A) and closer (B) views of a 59-year-old man with ectopic hidradenitis suppurativa presenting as a chronic tender, erythematous nodular plaque (black arrow) on his right posterior thigh.

Physical examination revealed a 4.0 × 3.5-cm tender, erythematous nodular plaque with peripheral scale on the right posterior thigh (Figure 1). The clinical differential diagnosis, based on the morphology of his lesion, included furuncle, carbuncle, abscess, and suppurative cellulitis. A bacterial culture of the purulent drainage was collected and treatment with oral cephalexin 500 mg four times a day and topical mupirocin 2% ointment was initiated.

He followed up three weeks later. A persistently tender, erythematous plaque on the right posterior thigh with newly formed sinus tracts was observed; scant drainage could be expressed after squeezing the lesion. Ectopic hidradenitis suppurativa was considered. A biopsy for pathologic evaluation and bacterial, fungal, and mycobacterial cultures was performed.

Microscopic examination of the tissue biopsy revealed suppurative inflammation with mixed inflammatory infiltrates. Apocrinitis was not observed; we speculate that this was due to the destruction of adnexal structures by the inflammatory process. Subsequently, the results of the tissue cultures for infectious pathogens were negative. Correlation of clinical and pathologic examination established the diagnosis of ectopic hidradenitis suppurativa.

The patient received 2 mL of 10 mg/mL of intralesional triamcinolone and was started on oral doxycycline monohydrate 100 mg twice daily, chlorohexidine gluconate washes daily, and topical 1% clindamycin solution twice daily. Due to the novel coronavirus 2019 pandemic and clinic closure, the patient was not seen for six months.

**Figure 2:**
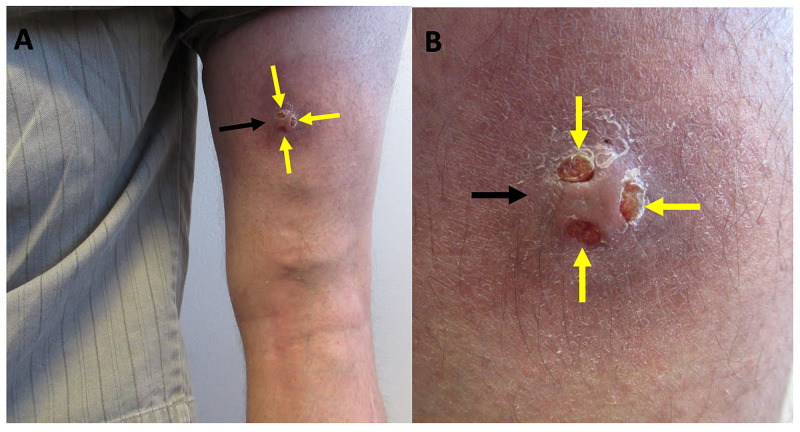
Clinical presentation of ectopic hidradenitis suppurativa at six-month follow-up. Distant (A) and closer (B) views of the nodular plaque of ectopic hidradenitis suppurativa on the right posterior thigh (black arrow). Residual sinus tracts (yellow arrows) are present following treatment with intralesional triamcinolone, oral doxycycline, and topical wound care.

At the time of follow-up, drainage had subsided and his associated pain had resolved. In addition, the lesion had decreased in size; however, residual sinus tracts persisted (Figure 2). The patient was content with the clinical improvement and did not want any additional treatment.

## Discussion

Hidradenitis suppurativa is a chronic, relapsing follicular-based inflammatory condition. The onset of hidradenitis suppurativa typically occurs between the ages of 18 and 39 years with a clear sex and racial predilection. In addition to family history, hidradenitis suppurativa is associated with female gender and Black race; furthermore, modifiable risk factors have been identified as smoking and obesity. Hidradenitis suppurativa has also been associated with autoimmune conditions, such as thyroid disease; however, hidradenitis suppurativa has not been previously associated with thyroid cancer, as seen in our patient [1,2].

Initial clinical presentation of hidradenitis suppurativa can range from scattered inflammatory nodules to severe, deep-seated abscesses with sinus tracts and malodorous, purulent discharge. These lesions classically occur in moist, intertriginous regions where apocrine glands are rich: the anogenital, axillary, inframammary, and inguinal regions. The Hurley clinical staging system can be used to better characterize the severity of hidradenitis suppurativa; in addition, treatment guidelines have been established for each of the three disease groups within the classification [1,2,13].

Uncommonly, hidradenitis suppurativa can occur in atypical regions where apocrine glands are scant or absent. Previously reported individuals with ectopic hidradenitis suppurativa had their lesions on the abdomen, amputated limb, caesarian scar, chest, dorsal foot, eyelids, knees, retroauricular region, and scalp (Table 1) [3-13]. Given the various affected regions and spectrum of clinical presentations, ectopic hidradenitis suppurativa can be very difficult to diagnose.

**Table 1:** Summary of the clinical features in reported individuals with ectopic hidradenitis suppurativa. CR, current report

Location	Clinical summary	Reference
Abdomen	A 54-year-old man without a previous history of hidradenitis suppurativa who was found to have ectopic hidradenitis suppurativa at the location of a massive abdominal hernia postulated to be secondary to mechanical stress of his waistband. Management of his hidradenitis suppurativa was not described.	[3]
Amputation stump	(1) A 44-year-old overweight man who smoked presented with recurrent inflammatory, deep-seated nodules, abscesses, and fistulas on his left lower limb amputation stump that resolved with surgery and topical medication. (2) A 55-year-old man with metabolic syndrome, Charcot-Marie-Tooth disease, and active tobacco use who presented with stump fistulas, abscesses, and scars restricted to his left lower limb amputation stump that improved with adalimumab. (3) A 41-year-old obese man who was an active smoker and had a personal history of hidradenitis suppurativa on his inner thigh, chest, and abdominal skin folds. He presented with recurrence of his hidradenitis suppurativa on the lower limb stump that failed to resolve with oral doxycycline. Surgical excision was planned; however, the outcome was not described.	[4,5]
Caesarian scar	A 33-year-old pregnant woman with history of hidradenitis suppurativa who had been managed on adalimumab until the end of her second trimester subsequently developed small nodules and inflammatory tracts on her caesarian scar two months after surgery that resolved with doxycycline, wound care, and resumption of adalimumab.	[6]
Chest	A 33-year-old obese woman who was an active smoker with an 18-year history of recurrent inflammatory nodules in the axillae, groin, pubic region, and chest corresponding to the lower edge of her bra developed hidradenitis suppurativa in both classical and atypical locations.	[7]
Dorsal foot	A 28-year-old man who smoked with a history of hidradenitis suppurativa presented with a painful, bleeding ulcer on his right dorsal foot and active hidradenitis suppurativa lesions on his femoral and perianal area that improved with surgical intervention.	[8]
Eyelids	A woman with a previous history of hidradenitis suppurativa who presented to her optometrist with ulcerative-like lesions that resembled herpetic blepharitis and were later determined to be ectopic hidradenitis suppurativa. Her management was not described.	[9,10]
Knee	A 60-year-old man who smoked with a history of type II diabetes mellitus who presented with multiple, painful, punched out ulcers on both of his knees with subsequent sinus tract formation that improved with adalimumab.	[11]
Neck	A 27-year-old healthy man with a 17-year history of hidradenitis suppurativa that predominantly occurred on the nape of his neck and bilateral axilla. His management was not described.	[12]
Posterior thigh	A 59-year-old man with a persistent, painful nodular plaque on his posterior thigh with subsequent sinus tract formation that improved with intralesional triamcinolone, oral doxycycline, and wound care.	CR
Retroauricular	A 49-year-old woman with a history of itchy, scaly lesions on both retroauricular regions who presented with erythematous nodules and sinus tracts that were diagnosed as ectopic hidradenitis suppurativa. Her management was not described.	[12]
Scalp	A 41-year-old obese man who smoked with recurrent atypical hidradenitis suppurativa on his posterior neck and scalp, preauricular cheek, lateral chest wall, and lateral hip with partial improvement on infliximab.	[13]

Both classic and ectopic hidradenitis suppurativa can be clinically diagnosed; pathologic confirmation is not always necessary to establish the diagnosis. Several dermatologic conditions can mimic ectopic hidradenitis suppurativa; therefore, making a timely diagnosis and initiating treatment can be challenging. In a retrospective chart review of patients with hidradenitis suppurativa in both classic and atypical locations, it was revealed that the mean time from initial onset to proper diagnosis was 12 years [14]. The differential diagnosis for hidradenitis suppurativa includes acne vulgaris, carbuncle, Crohn’s disease, folliculitis, furuncle, granuloma inguinale, pilonidal cyst, and pyoderma gangrenosum [1].

While the pathogenesis of hidradenitis suppurativa is not entirely understood, the leading theory suggests epithelial hyperkeratosis causes follicular occlusion, which leads to dilation, rupture, and spilling of the follicular contents and a resultant robust inflammatory response in the surrounding dermis. This phenomenon usually occurs in apocrine-rich areas; however, apocrinitis is not the primary event but rather the result of local inflammation secondary to follicular occlusion and rupture. In ectopic hidradenitis suppurativa, the initial event causing hyperkeratosis and subsequent follicular plugging has been postulated to be caused by mechanical stress; our patient developed ectopic hidradenitis suppurativa on his right posterior thigh which was a high friction point for him when seated [4,7,9,15,16].

The robust initial dermal inflammation with subsequent abscess and sinus tract formation in hidradenitis suppurativa has been postulated to be orchestrated by two key cytokines, interleukin-17 (IL-17) and tumor necrosis factor alpha (TNF-alpha); indeed, measured levels of TNF-alpha in affected skin and IL-17 in serum directly correlate with disease severity. Other suspected inflammatory markers in the pathogenesis of hidradenitis suppurativa include IL-1, IL-6, IL-12, and IL-23. Based on the identification of numerous cytokines, agents that block these inflammatory markers have been reported in the management of hidradenitis suppurativa [2,17].

Management of hidradenitis suppurativa can be difficult and is often individualized; typically, it begins with topical and/or oral antibiotics, topical wound care, intralesional corticosteroids, and lifestyle improvement that includes dietary modification, weight loss, and smoking cessation. Currently, adalimumab, an anti-TNF agent, is the only Food and Drug Administration-approved biologic agent for the treatment of hidradenitis suppurativa in patients older than 12 years. Previous trials have demonstrated the efficacy of adalimumab in the treatment of hidradenitis suppurativa to range from 41% to 58%. Biologic agents targeting IL-17 (secukinumab) and IL-12/IL-23 (guselkumab) have also been used by individual patients with varying efficacy to treat hidradenitis suppurativa; however, multi-center, randomized, double-blind studies of these medications are currently ongoing to assess efficacy over a larger population with hidradenitis suppurativa. To date, drug trials with concurrent use of adalimumab with either secukinumab or guselkumab have not been performed [1,17-19].

For patients with severe, medication-resistant hidradenitis suppurativa, surgery may be considered as a possible treatment modality. Conservative surgical interventions include simple drainage, limited excision, and deroofing or marsupialization of sinus tracts with healing by secondary intention; these procedures are associated with a low morbidity but a high rate of recurrence. Radical excision of the affected area has the highest probability of complete resolution; however, recurrence has been observed due to inadequate excision or unusually wide apocrine gland distribution [9].

## Conclusions

Hidradenitis suppurativa is a recurrent, painful follicular-based inflammatory condition that classically occurs in moist, intertriginous apocrine-rich areas but may also occur in atypical locations. We describe a man who presented with a tender, erythematous nodular plaque with subsequent sinus tract formation on his right posterior thigh that was determined to be ectopic hidradenitis suppurativa. Based upon the broad clinical presentation and difficultly establishing a timely diagnosis of ectopic hidradenitis suppurativa, the condition should be considered in patients who present with painful, erythematous nodular lesions in high friction regions that have failed to improve with conventional treatment.

## References

[1] Lee E, Alhusayen R, Lansang P, Shear N, Yeung J (2017). What is hidradenitis suppurativa?. Can Fam Physician.

[2] Goldburg S, Strober B, Payette M (2020). Hidradenitis suppurativa: epidemiology, clinical presentation, and pathogenesis. J Am Acad Dermatol.

[3] de Vita V, Fabbrocini G (2018). Mechanical stress as a cause of hidradenitis suppurativa: a lesson from a patient with a monster hernia. Acta Dermatovenerol Croat.

[4] de Winter K, van der Zee HH, Prens EP (2012). Is mechanical stress an important pathogenic factor in hidradenitis suppurativa?. Exp Dermatol.

[5] Kluger N, Guillem P, Kivivuori M, Isoherranen K (2020). Hidradenitis suppurativa or hidradenitis suppurativa-like lesions located on amputation stumps? Description of 2 cases. Skin Appendage Disord.

[6] Darch KM, Holland TL, Spelman LJ (2020). Hidradenitis suppurativa recurrence in a caesarean scar. Case Rep Obstet Gynecol.

[7] Boer J, Mihajlovic D (2016). Boils at frictional locations in a patient with hidradenitis suppurativa. Acta Dermatovenerol Croat.

[8] Rondags A, Diercks GF, Werker PMN, Jonkman MF, Horváth B (2017). Ectopic hidradenitis suppurativa on the dorsal foot of a road maker. JAAD Case Rep.

[9] Slade DEM, Powell BW, Mortimer PS (2003). Hidradenitis suppurativa: pathogenesis and management. Br J Plast Surg.

[10] Mastrota K (2018). Hidradenitis suppurativa masquerades as blepharitis. Optom Times.

[11] Gosnell HL, Sharghi K, Pickard C, Grider DJ (2020). Hidradenitis suppurativa at the knees. Dermatol Online J.

[12] Syed ZU, Hamzavi IH (2011). Atypical hidradenitis suppurativa involving the posterior neck and occiput. Arch Dermatol.

[13] Naasan H, Affleck A (2015). Atypical hidradenitis suppurativa. Clin Exp Dermatol.

[14] Mebazaa A, Ben Hadid R, Cheikh Rouhou R (2009). Hidradenitis suppurativa: a disease with male predominance in Tunisia. Acta Dermatovenerol Alp Pannonica Adriat.

[15] von Laffert M, Stadie V, Wohlrab J, Marsch WC (2011). Hidradenitis suppurativa/acne inversa: bilocated epithelial hyperplasia with very different sequelae. Br J Dermatol.

[16] Boer J (2017). Should hidradenitis suppurativa be included in dermatoses showing koebnerization? Is it friction or fiction?. Dermatology.

[17] Narla S, Lyons AB, Hamzavi IH (2020). The most recent advances in understanding and managing hidradenitis suppurativa. F1000Res.

[18] Goldburg SR, Strober BE, Payette MJ (2020). Hidradenitis suppurativa: current and emerging treatments. J Am Acad Dermatol.

[19] Kimball A, Okun M, Williams D (2016). Two phase 3 trials of adalimumab for hidradenitis suppurativa. N Engl J Med.

